# High-Efficiency, Dual-Band Beam Splitter Based on an All-Dielectric Quasi-Continuous Metasurface

**DOI:** 10.3390/ma14123184

**Published:** 2021-06-09

**Authors:** Jing Li, Yonggang He, Han Ye, Tiesheng Wu, Yumin Liu, Xuyi He, Jing Li, Jie Cheng

**Affiliations:** 1 State Key Laboratory of Information Photonics and Optical Communications, University of Posts and Telecommunications, Beijing 100876, China; Li_jing@bupt.edu.cn (J.L.); Han_ye@bupt.edu.cn (H.Y.); 2 College of Information and Communication Engineering, Guilin University of Electronic Technology, Guilin 541004, China; tieshengw@guet.edu.cn; 3 School of Mechanical Engineering, Nanjing University of Science & Technology, Nanjing 210094, China; wangyuwy@bupt.edu.cn; 4 School of Electronics and Internet of Things, Chongqing College of Electronic Engineering, Chongqing 401331, China; hexuyi0920@sina.com; 5 State Grid Information & Telecommunication Branch, Beijing 100761, China; hjanevan@bupt.edu.cn (J.L.); chengjie@sgcc.com.cn (J.C.)

**Keywords:** beam splitter, quasi-continuous metasurface, refraction, dual-band

## Abstract

Metasurface-based beam splitters attracted huge interest for their superior properties compared with conventional ones made of bulk materials. The previously reported designs adopted discrete metasurfaces with the limitation of a discontinuous phase profile. In this paper, we propose a dual-band beam splitter, based on an anisotropic quasi-continuous metasurface, by exploring the optical responses under x-polarized (with an electric field parallel to the direction of the phase gradient) and y-polarized incidences. The adopted metasurface consists of two identical trapezoidal silicon antenna arrays with opposite spatial variations that lead to opposite phase gradients. The operational window of the proposed beam splitter falls in the infrared and visible region, respectively, for x- and y-polarized light, resulting from the different mechanisms. When x-polarized light is incident, the conversion efficiency and total transmission of the beam splitter remains higher than 90% and 0.74 within the wavelength range from 969 nm to 1054 nm, respectively. In this condition, each array can act as a beam splitter of unequal power. For y-polarized incidence, the maximum conversion efficiency and transmission reach approximately 100% and 0.85, while the values remain higher than 90% and 0.65 in the wavelength range from 687 nm to 710 nm, respectively. In this case, each array can be viewed as an effective beam deflector. We anticipate that it can play a key role in future integrated optical devices.

## 1. Introduction

Beam splitters are optical components that divide a beam of light into two or more beams. They are indispensable components in several optical and photonic applications, such as interferometers, photo cameras, optical communications and laser systems. So far, the reported beam splitters are primarily implemented based on the following structures, such as photonic crystals [1,2], gratings [3,4], directional couplers [5], multimode interference (MMI) structures [6], and metamaterials [7,8,9,10]. However, the beam splitters made from conventional optical components are bulky and heavy, which makes it difficult to integrate them with other optical devices and limits their application in miniature photonic circuits. Recently, the development of metasurfaces provides new solutions to these problems. A metasurface, a type of artificial material with sub-wavelength thickness, enables the manipulation of the phase [11,12,13,14,15], amplitude [16,17,18] and polarization [19,20,21] of the light beams at will. Compared to natural materials, metasurfaces possess several advantages, such as smaller size, lower loss, and higher flexibility. Thanks to these advantages, the metasurface-based beam splitter (MBBS) has received significant attention in recent years.

**Figure 1:**
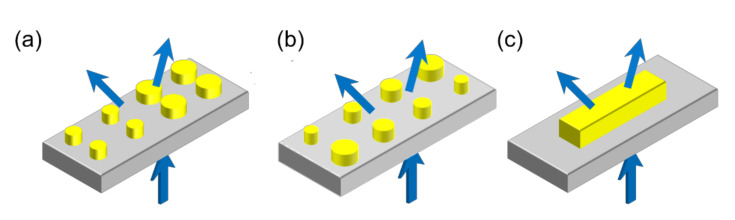
(**a**) Beam splitter based on a metasurface composed of binary unit cell arrays. (**b**) Beam splitter based on a discrete metasurface composed of two antenna arrays with opposite phase gradients. (**c**) Beam splitter based on a uniform metasurface.

At present, there are mainly three types of beam splitters utilizing metasurface implementation [22,23,24,25,26]. The first kind of beam splitter is based on a discrete metasurface which is composed of a periodic array of binary unit cells with π phase difference between adjacent cells [22,23], as shown in Figure 1a. However, since the phase delay induced by the nanoantenna is sensitive to wavelength, it is difficult to find two antennas with π phase difference in a wide band, especially for a dielectric metasurface. Therefore, the dielectric metasurface designed by this method can only exhibit efficient equal-power beam splitting in a narrow spectral range. The second kind of beam splitter is based on a discrete metasurface which consists of two antenna arrays with opposite phase gradients, and each of the arrays can achieve complete 2π phase control [24,25], as shown in Figure 1b. In these arrays, multiple different antennas are chosen to cover the full 0 to 2π phase range. Even if the MBBSs designed using this method always possess equal-power splitting characteristics, it is still difficult to maintain high conversion efficiency in a wide band. The two kinds of MBBSs above have the disadvantages of a narrow band and low efficiency. The third kind of beam splitter can solve these problems [26]. This beam splitter is based on a uniform metasurface, which is only composed of a square array of nanoblocks, as shown in Figure 1c. The beam splitter possesses the characteristics of a broad-band, high efficiency and large angle at the same time. However, the design has high requirements on the polarization of the incident light.

In this paper, we propose an equal-intensity beam splitter based on a quasi-continuous metasurface, which is composed of two identical trapezoidal antenna arrays with opposite directions. Compared with the discrete metasurface, the quasi-continuous metasurface possesses the advantages of easy fabrication, high efficiency, wide bandwidth and simple structure. Moreover, the structural anisotropy of the trapezoidal building blocks also provides a way to realize the dual-band performance. The optical response and the operating wavelength of the beam splitter under x- and y-polarized incidences are different, due to the structural anisotropy of the trapezoidal antennas. For x-polarized normal incidence, the conversion efficiency and total transmission remain higher than 90% and 0.74 within the wavelength region from 969 nm to 1054 nm, respectively. For y-polarized normal incidence, the conversion efficiency and total transmission remain higher than 90% and 0.65, respectively, within the wavelength region from 687 nm to 710 nm.

## 2. Materials and Methods

**Figure 2:**
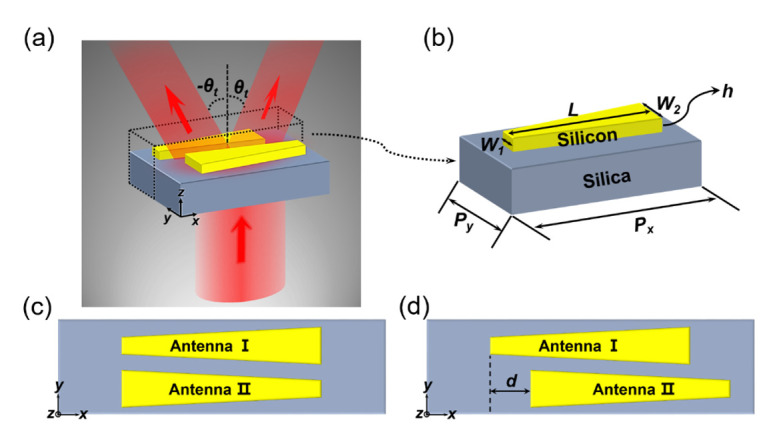
(**a**) Schematic of the proposed metasurface-based beam splitter. (**b**) The three-dimensional structure diagram of a supercell, consisting of only one trapezoidal silicon antenna placed on the silica substrate. The design procedure of the beam splitter (vertical view). In a supercell, (**c**) these two identical antennas that form a supercell are aligned in the y-direction, (**d**) The antenna II is shifted by d along the positive x-axis with respect to antenna I.

The schematic diagram of the proposed metasurface-based beam splitter is shown in Figure 2a. In order to realize beam splitting, we introduced two opposite phase gradients in the metasurface by adopting two identical quasi-continuous dielectric antenna arrays with opposite spatial variation. Firstly, we designed a nanoantenna array, the supercell of which consists of only one trapezoidal silicon antenna. Figure 2b shows the three-dimensional structure diagram of the supercell resting on the silica substrate. The period of the supercell along the x- and y-directions was optimized to be Px = 1360 nm and Py = 190 nm, respectively. The height h of the antenna was set as 240 nm, the length L is 806 nm, and the width varied linearly from W_1_ = 66 nm to W_2_ = 154 nm. Finally, we added an identical array with opposite spatial arrangement into the array designed above, to compose the metasurface. As shown in Figure 2c, the supercell of the metasurface is composed of two identical trapezoid-shaped silicon antennas, I and II. The width of antenna I increases along the positive x-axis, while the other antenna holds the opposite spatial arrangement. In addition, as depicted in Figure 2d, when antenna II is introduced, we can move it relative to antenna I, which increases the degree of freedom of the design. As antenna II moves, the interaction between these two antennas will change, which will affect the optical response of the metasurface. Therefore, we took the moving distance d of antenna II relative to antenna I as a key factor to optimize the beam splitting effect of the metasurface. The relative moving distance d is defined as a positive (negative) value when antenna II is shifted toward the positive (negative) x-axis relative to antenna I. In this paper, we set the relative moving distance as 200 nm to achieve efficient beam splitting of x- and y-polarized incident light. We adopted the Lumerical finite-difference time-domain (FDTD) Solutions package to investigate the optical properties of this design. Periodic boundary conditions were applied in both x- and y-directions, and perfectly matched layers were used along the z-direction. The optical constants of silicon are taken from [27] and the refractive index of silica is 1.45. The linearly polarized plane wave is normally incident from the bottom of the silica substrate. Due to the anisotropy of the trapezoidal structure, the optical response of the quasi-continuous metasurface was sensitive to the polarization, so the operating wavelength of x-polarized light is different from that of y-polarized light. The wavelength range of x-polarized incident light is from 960 nm to 1060 nm, while the wavelength region of y-polarized light is 680 nm to 720 nm.

## 3. Results

In this section, firstly, we analyze the optical response of the quasi-continuous metasurface composed of antenna I array under x- and y-polarized incidence. In order to achieve beam splitting, we adopted two identical trapezoidal antenna arrays (antenna I array and antenna II array) with opposite spatial variation to compose the metasurface. We investigate the influence of the moving distance d of antenna II relative to antenna I on the beam-splitting performance of the metasurface under x- and y-polarized incidence, respectively, and determine the structure of MBBS. Finally, we analyze the beam-splitting performance of the MBBS.

### 3.1. The Optical Response of the Quasi-Continuous Metasurface Composed of Antenna I Array

**Figure 3:**
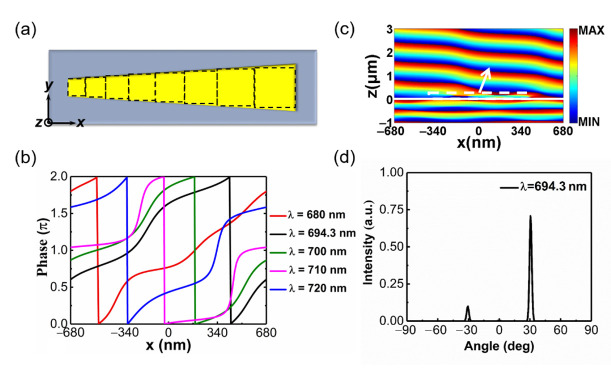
(**a**) Vertical view of a supercell of the quasi-continuous phase-gradient metasurface. (**b**) Phase response for y-polarized plane wave incidence with wavelengths of 680, 694.3, 700, 710 and 720 nm. (**c**) Ey field pattern in the x-z plane and (**d**) normalized far-field intensity distributions of the transmitted beams at the wavelength of 694.3 nm.

Firstly, we explore the optical responses of the quasi-continuous metasurface composed of antenna I array under x- and y-polarized incidences. As shown in Figure 3a, in order to understand the phase response of this metasurface, the trapezoidal nanoantenna can be approximately viewed as composed of multiple discrete geometry-varied blocks [28]. Details of the geometric parameters of these building blocks and the design of the trapezoidal nanoantenna are supplied in Appendix A. When these nanoblocks are combined into a trapezoidal antenna, we arrange them compactly from small to large, instead of filling the gaps between them [28]. In this process, the spacing between adjacent nanoblocks gradually decreases, resulting in the increase of coupling between them. Therefore, there are differences in optical response between the quasi-continuous metasurface and the original discrete metasurface, especially for x-polarized incident light. The corresponding phase response and far-field distribution of the quasi-continuous metasurface under y- and x-polarized incidences are as follows.

We numerically simulate the phase response of quasi-continuous phase-gradient metasurface under the normal incidence of a y-polarized plane wave. The incident wavelength of the plane wave ranges from 680 nm to 720 nm. To illustrate it more intuitively and clearly, we depict the transmission phase shift curve at various wavelengths in Figure 3b. It can be found that the 2π phase shift with nearly linear variation can always be achieved within this band. The incident light can be redirected by the metasurface with 2π phase control according to the generalized Snell’s law [11]:

(1) $$n_{t}{\sin\theta }_{t}-n_{i}{\sin\theta }_{i}=\frac{\lambda_{0}}{2\pi }\frac{d\varphi }{\mathrm{dx}}$$

where θ_i_ is the incident angle, n_i_ and n_t_ are the refractive indices of the incident and transmitted medium, respectively, λ_0_ is the wavelength in vacuum, dφ is the phase difference between adjacent units along the x-direction, and dx is the period of the unit along the x-direction. The angle of emergence θ_t_ can be freely modulated by adjusting the phase gradient dφ/dx. The transmitted electric field depicted in Figure 3c illustrates that the normally incident light is redirected by the metasurface. According to Equation (1), the anomalous transmitted beam is deflected at an angle of 30.7° for a wavelength of 694.3 nm. The far-field distribution at the wavelength of 694.3 nm is depicted in Figure 3d. The transmitted light mainly propagates along the angle of 30.7°, which is consistent with the theoretical deflected angle. At the wavelength of 694.3 nm, it is acceptable that the −1 order diffraction is not well suppressed. Since we ultimately want to achieve beam splitting, perfect suppression of the zero-order diffraction is what we need.

**Figure 4:**
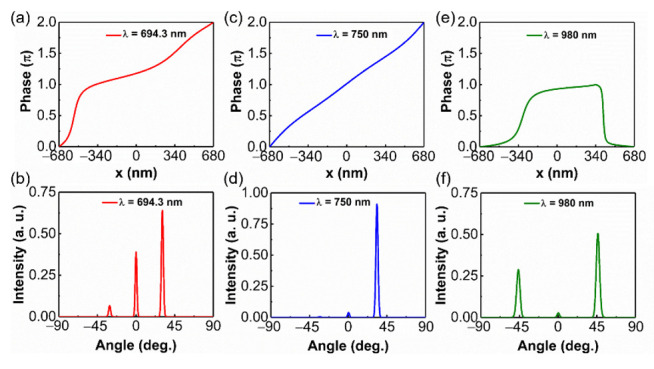
Phase response for x-polarized plane wave incidence at the wavelengths of (**a**) 694.3 nm, (**c**) 750 nm, (**e**) 980 nm. Far-field diffraction patterns for x-polarized plane wave incidence at the wavelengths of (**b**) 694.3 nm, (**d**) 750 nm, (**f**) 980 nm.

The optical response of the quasi-continuous phase-gradient metasurface under the incidence of the x-polarized plane wave is also investigated. Figure 4 shows the phase response and far-field profile of the metasurface at various wavelengths. As shown in Figure 4a, although the 2π phase coverage is achieved at a wavelength of 694.3 nm, the linearity of the phase change along the x-position is not good, which leads to an undesirable anomalous refraction effect. It is also confirmed by the far-field pattern in Figure 4b, where only a part of the incident light transfers into the anomalous refraction direction. With the widening of the wavelength, we find that the working wavelength of the metasurface exhibiting an abnormal refraction effect under x-polarized incidence is 750 nm. The phase response and corresponding far-field distribution are depicted in Figure 4c,d. A full 2π phase shift with a constant gradient is realized, resulting in the near-perfect suppression of other undesired diffraction orders. Unfortunately, the operating band of efficient anomalous refraction is only 4 nm. The diffraction efficiency is a key factor of the beam deflector; it is defined as the power of the deflected beam in the desired +1 diffraction order, normalized to total transmission power. In our paper, we define that when the diffraction efficiency is higher than 90%, the metasurface exhibits efficient anomalous refraction, and the corresponding wavelength is viewed as the operating wavelength. Thus, using two arrays with opposite phase gradients, both of which can realize efficient anomalous refraction, to achieve beam splitting [24] is not suitable for x-polarized incidence. Here, we adopt another mechanism to accomplish beam splitting. As shown in Figure 4e, the phase change of trapezoidal antenna along the x-position is small at the wavelength of 980 nm. The antenna can be approximately regarded as an element with π phase discontinuity from the surface, leading to a phase profile of 0-π-0. The transmitted light propagates along the direction of +1 and −1 diffraction orders, but the intensity of these two beams is different, as depicted in Figure 4f. Under x-polarized incidence, the metasurface can be used as an unequal-power beam splitter in the wavelength range from 969 nm to 1054 nm. Finally, by introducing the same array with the opposite spatial arrangement, the difference between the intensity of +1 and −1 diffraction order can be compensated, thus equal-power beam splitting can be realized.

### 3.2. Influence of the Relative Moving Distance 

As mentioned above, in order to achieve beam-splitting, we adopted two identical trapezoidal antenna arrays with opposite spatial variation to compose the metasurface. For y-polarized incidence, each array can be viewed as an effective beam deflector. When x-polarized light is incident, each array can act as an unequal-power beam splitter. The relative movement between two antenna arrays will affect the optical response of the metasurface. Here, we investigate the influence of the moving by distance d of antenna II relative to antenna I on the beam-splitting performance of the metasurface. As shown in Figure 2d, the relative moving distance d can be changed from –554 nm (−|Px-L|) to 554 nm (Px-L). Two indexes, conversion efficiency and transmission intensity, are used to evaluate the beam-splitting performance. We define the conversion efficiency *η* of the metasurface as:

(2) $$\eta =\frac{I_{+1}+I_{-1}}{I}\times 100\%$$

where *I*_+1_ and *I*_−1_ are the intensity of +1 and −1 diffraction order, respectively, and *I* is the total transmission intensity.

**Figure 5:**
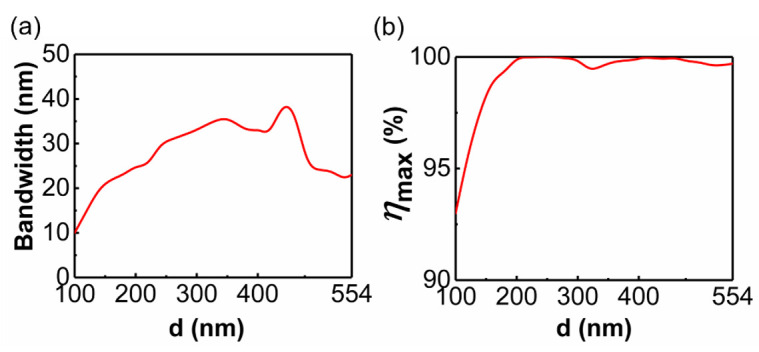
The simulated optical performances for the metasurface with different d under the y-polarized incidence. (**a**) The bandwidth of the metasurface operating as an efficient beam splitter. The band with a conversion efficiency higher than 90% is regarded as the operating band. (**b**) The highest conversion efficiency *η*_max_ in the corresponding wave band.

The simulated optical performances for the metasurface with different relative moving distance d under the incidence of y-polarized light are displayed in Figure 5. We determine that the metasurface exhibits efficient beam-splitting when the conversion efficiency is greater than 90%, and take the corresponding wavelength as the operating wavelength. As shown in Figure 5a, when the relative moving distance changes from 100 nm to 554 nm, the metasurface can always achieve effective beam-splitting in a wide bandwidth. Particularly, the bandwidth reaches the maximum value of 39 nm when the shifting distance is 440 nm. When the moving distance is less than 100 nm, the metasurface can only exhibit effective beam-splitting at a certain wavelength, so the corresponding results are not displayed. The maximum conversion efficiency *η*_max_ in the corresponding band for different relative moving distances is depicted in Figure 5b. It will always reach above 99% when the moving distance d is greater than 180 nm.

**Figure 6:**
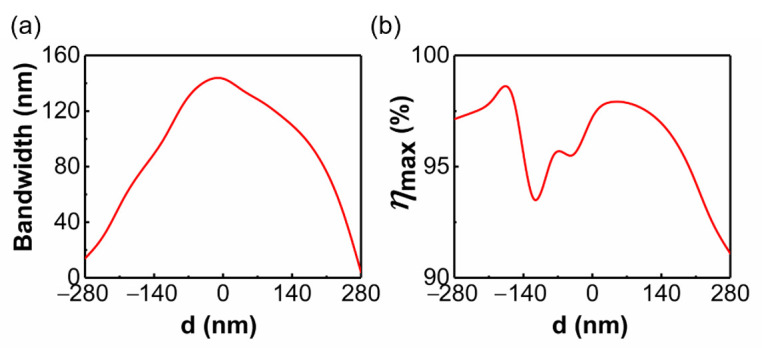
The simulated optical performances for the metasurface with different *d* under the x-polarized incidence. (**a**) The bandwidth of the metasurface operating as an efficient beam-splitter. The band with a conversion efficiency higher than 90% is regarded as the operating band. (**b**) The highest conversion efficiency *η*_max_ in the corresponding wave band.

As shown in Figure 6a, for x-polarized incidence, the metasurface can always achieve effective beam splitting within a wide bandwidth when the relative moving distance varies from −280 nm to 280 nm. Particularly, the operating bandwidth remains around 140 nm within the relative moving distance d ranging from −40 nm to 40 nm. It should be noted that there may be multiple bands with a conversion efficiency higher than 90% for these metasurfaces, and here we chose the longest band to more accurately compare optical performance. The maximum conversion efficiency *η*_max_ in the corresponding band for different moving distances is depicted in Figure 6b. It is sensitive to the relative moving distance, and reaches the maximum value of 99.8% when d is −160 nm. By comprehensively comparing the above data, we finally chose the metasurface with a relative moving distance of 200 nm as the beam splitter.

### 3.3. The Optical Performance of the Proposed Beam Splitter

**Figure 7:**
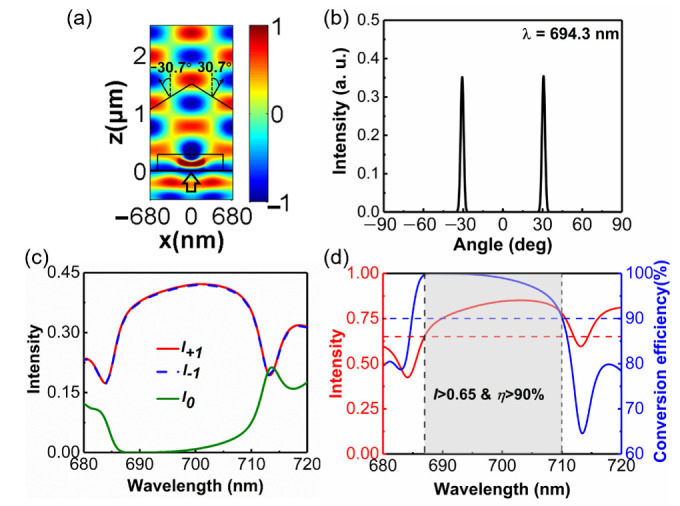
(**a**) Ey field pattern in the x-z plane under y-polarized normal incidence at a wavelength of 694.3 nm. (**b**) Normalized far-field intensity distributions of the transmitted beams. (**c**) Intensity of +1, 0, and −1 diffraction orders within the wavelength region from 680 nm to 720 nm. (**d**) Transmission intensity and conversion efficiency of the transmitted beam for y-polarized incidence in the wave range from 680 nm to 720 nm. The red dashed line indicates that the transmission is 0.65, the blue dashed line represents the conversion efficiency is 90%. The transmitted angle is defined as the positive (negative) value in the right (left) side of the normal.

When a y-polarized plane wave with a wavelength of 694.3 nm is normally incident to the metasurface, the transmitted electric field and far-field distributions are depicted in Figure 6a,b, respectively. The metasurface divides the incident beam into two parts, which transmit along the left and right sides of the normal direction at the same angle of 30.7° in the x-z plane. Due to the interaction between these two light beams propagating along different directions, the wavefront is no longer planar and uniform like that of a single beam, as shown in Figure 7a. From Figure 7b, it can be seen that there are only three diffraction orders: −1, 0, and +1, which agrees well with theoretical calculations [29]. The intensity of the two beams propagating along the + 1 and −1 diffraction orders is about the same value of 0.401. The zero-diffraction order is well suppressed, and its intensity is only 0.0008. Hence, the designed metasurface operates as a high-efficiency beam splitter with equal power at the wavelength of 694.3 nm. In order to study the bandwidth of the metasurface as an efficient beam splitter, we simulate the optical performance within the wavelength region from 680 nm to 720 nm. Figure 7c depicts the intensities of +1, 0 and −1 diffraction orders and the total transmission intensity within the wavelength range from 680 nm to 720 nm. The intensities of the +1 and −1 diffraction orders of the designed metasurface are almost the same at any wavelength. The total transmission remains higher than 0.65 within the wavelength region 687 nm to 710 nm, and reaches the maximum value of 0.85 at the wavelength of 703 nm. The suppression of other diffraction orders is also an important performance of the beam splitter. The result depicted in Figure 7d shows that conversion efficiency remains above 90% within the wavelength region from 687 nm to 710 nm.

**Figure 8:**
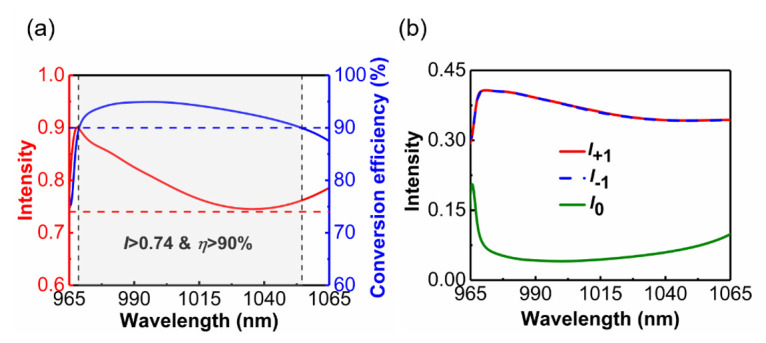
(**a**) Transmission intensity and conversion efficiency of the transmitted beam for x-polarized incidence in the wave range from 965 nm to 1065 nm. The red dashed line indicates that the transmission is 0.74, the blue dashed line represents that the conversion efficiency is 90%. (**b**) Intensity of +1, 0, and −1 diffraction orders within the wavelength region from 965 nm to 1065 nm.

Figure 8a shows the transmission intensity and conversion efficiency of the metasurface-based beam splitter within the wavelength region from 965 nm to 1065 nm. The red and blue dashed lines represent an intensity of 0.74 and a conversion efficiency of 90%, respectively. It can be seen that the conversion efficiency and transmission remain higher than 90% and 0.74, respectively, within the wavelength region from 969 nm to 1054 nm (the band corresponding to the gray area). The conversion efficiency reaches the maximum value, 95%, at the wavelength of 996 nm, and the corresponding transmission is 0.818. As shown in Figure 8b, the intensities of +1 and −1 diffraction orders are approximately the same within this band. Moreover, when the wavelength is larger than 1105 nm, the conversion efficiency of the metasurface is less than 40%. We consider that the metasurface can no longer be used as a beam splitter in this case. Therefore, we take 1105 nm as the limiting wavelength for realization of the beam-splitting function. The splitting angle between the two beams is 108.68° at a wavelength of 1105 nm.

## 4. Discussion

**Figure 9:**
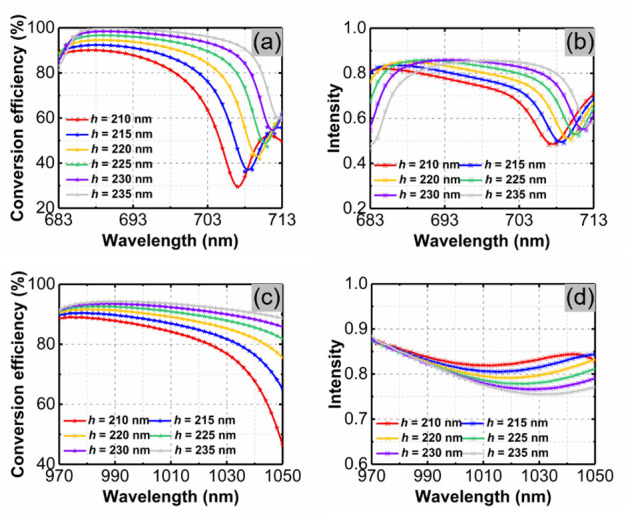
(**a**) Conversion efficiency and (**b**) transmission intensity of metasurfaces with different thicknesses under the incidence of y-polarized light. (**c**) Conversion efficiency and (**d**) transmission intensity of metasurfaces with different thicknesses under the incidence of x-polarized light.

It is well known that in these types of experiments, the metasurface with a lower aspect ratio is easier to fabricate. Here, we kept the other geometric parameters unchanged and reduced the thickness of the metasurface to explore the tolerance of beam-splitting characteristics to aspect ratio. Figure 9a,b show the conversion efficiency and transmission intensity of the metasurfaces with different thicknesses under the incidence of y-polarized light, respectively. When the thickness is 210 nm, the maximum conversion efficiency of the metasurface is less than 90%. As the thickness increases, the band of the metasurface that can realize efficient beam splitting gradually widens and the operating wavelength redshifts. When the thickness of the metasurface changes from 215 nm to 240 nm, we can always find a band in which the conversion efficiency is higher than 90% and the transmittance is greater than 0.7. For x-polarized incidence, these two optical performances of metasurfaces with different thicknesses are depicted in Figure 9c,d. It can be found that, in this condition, the effect of metasurface thickness on optical properties is consistent with that of y-polarized incidence. When the thickness increases from 215 nm to 240 nm, the working band of the metasurface becomes wider, and the transmission intensity is always higher than 0.7 in the corresponding band. Therefore, in the process of reducing its thickness to 215 nm, the metasurface can always achieve efficient beam splitting for x-polarized and y-polarized incidences.

**Figure 10:**
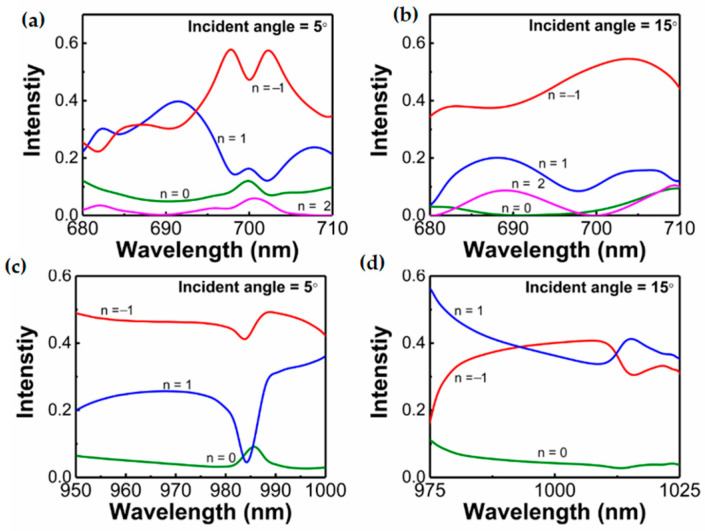
Intensity of each diffraction order when y-polarized light is incident at an angle of (**a**) 5° and (**b**) 15°. Intensity of each diffraction order when x-polarized light is incident at an angle of (**c**) 5° and (**d**) 15°.

The beam-splitting performances of the metasurface under normal incident are analyzed above. Here, we take the optical response of the metasurface at the incident angles of 5° and 15° as examples to investigate the performance of the proposed device at oblique incidence. Figure 10a,b depict the intensity of each diffraction order when y-polarized light is incident on the metasurface at an angle of 5° and 15°, respectively. 

It can be seen that there are four diffraction orders. This phenomenon can be explained by the modified diffraction equation [29]:

(3) $$\sin\theta_{t}-\sin\theta_{i}=(m_{0}+1)\frac{\lambda_{0}}{\eta }=m\frac{\lambda_{0}}{\eta }$$

where θ_t_ is the anomalous refraction angle, θ_i_ is the incident angle, λ_0_ is the wavelength in vacuum, *η* is the period of the supercell, m_0_ is the conventional diffraction order, m is the actual diffraction order of the metasurface. When the incident beam is along the normal direction, the incident angle θ_i_ is 0, according to Equation (3), the outgoing beams on the left and right sides are symmetrical about the normal in the x-z plane. However, this symmetry will be broken when the light is incident obliquely. In this case, the value of θ_i_ is no longer 0. It can be found that the angle between the emitted light along both sides of the normal and the normal is no longer equal. Here, we take the optical response of the metasurface at the incident angles of 5° and 15° as examples to investigate the performance of the proposed device at oblique incidence. Figure 10a,b depict the intensity of each diffraction order when y-polarized light is incident on the metasurface at an angle of 5° and 15°, respectively. As shown in Figure 10a, when the incident angle of y-polarized light is 5°, the intensities of −1 and +1 diffraction orders are no longer the same, and the metasurface does not significantly inhibit the light of −2 and 0 diffraction orders. In the wavelength range of 695 nm to 70 nm, the intensity of −1 diffraction order is obviously higher than that of other diffraction orders. Figure 10b shows that the light propagating along the −1 diffraction order accounts for the largest proportion of the total transmitted light, when the y-polarized light is incident at an angle of 15°. Figure 10c,d depict the intensities of +1, 0 and −1 diffraction orders when x-polarized light is incident on the metasurface at an angle of 5° and 15°, respectively. In both cases, the 0 diffraction order is not effectively suppressed. Thus, when x-polarized or y-polarized light is obliquely incident, the proposed metasurface can no longer effectively suppress the undesirable diffraction order, and the intensity of −1 and +1 diffraction order is no longer the same.

In order to show the advantages of the splitter, based on the quasi-continuous metasurface, as shown in Table 1, we compared the performance of our proposed beam splitter with the previously reported ones based on discrete dielectric metasurfaces in the visible and near-infrared range. In [22], the beam splitting is realized by adopting a metasurface composed of a periodic array of binary cells with a π phase difference between adjacent ones. At the operating wavelength of 532 nm, the splitter exhibits an equal-power characteristic, and its conversion efficiency and transmission are 92% and 0.9, respectively. However, the ratio of the intensities of the +1 and −1 diffraction orders is sensitive to the wavelength. In [24], a beam splitter is implemented by a metasurface comprising two discrete cylinder arrays with opposite phase gradients. In this case, the power of each diffraction order of +1 and −1 is always equal for any wavelength. The total transmission is lower than 0.6 at the operating wavelength of 800 nm, while the intensity of the zeroth-order is not mentioned. The optical performances of the above two beam splitters are not very sensitive to polarization. In our design, the beam splitter operates in different wave bands for x-and y-polarization incidences. For x-polarized light, the conversion efficiency and transmission are 95% and 0.818, respectively, at a wavelength of 996 nm. For y-polarized light, the operating band moves to a visible region. The conversion efficiency and transmission are approximately 100% and 0.803, respectively, at a wavelength of 694.3 nm. The equal-power characteristic is valid for both x- and y-polarized incidences at an arbitrary wavelength.

## 5. Conclusions

In summary, we have proposed an equal-power and dual-band metasurface-based beam splitter. In the supercell of the metasurface, two identical trapezoidal antennas with opposite spatial arrangement are adopted, and the two antennas are moved 200 nm relative to each other along the x-direction. Due to the anisotropy of the metasurface, its optical properties are different for x-polarized and y-polarized incidences. For the y-polarized normal incidence, the conversion efficiency and total transmission remain higher than 90% and 0.65, respectively, within the wavelength region from 687 nm to 710 nm. For the x-polarized light, the conversion efficiency is higher than 90% accompanied by the transmission greater than 0.74 in the 85 nm bandwidth, respectively. We expect that the proposed beam splitters can be applied in many compact integrated devices.

## Figures and Tables

**Table 1:** Optical performances of our proposed beam splitter and previously reported splitters based on discrete dielectric metasurfaces.

Splitter	Material	Wavelength	Conversion Efficiency	Transmission	Propagation Angle
Ozer et al. [22]	TiO_2_	532 nm	92%	0.9	±46.8°
Zhang et al. [24]	lithium niobate	800 nm	-	<0.6	±12.17°
Our paper	silicon	996 nm (x-pol.)	95%	0.818	±47.1°
		694.3 nm (y-pol.)	≈100%	0.803	±30.7°

**Figure A1:**
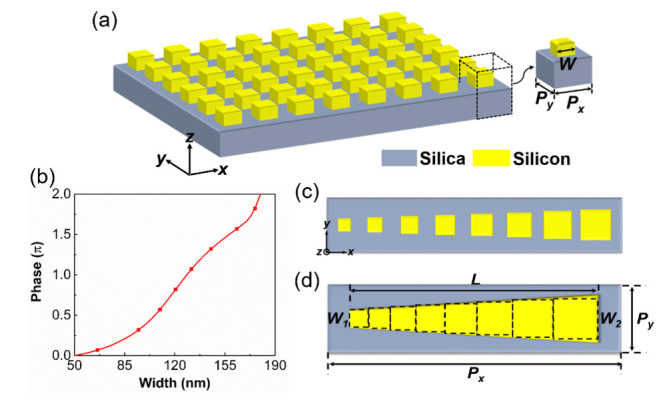
(**a**) Schematic of the homogeneous metasurface composed of silicon blocks rested on silica substrate. (**b**) The phase of the transmitted light as the function of the width *W* of the silicon block at a wavelength of 694.3 nm. (**c**) Vertical view of a supercell composed of eight different blocks. (**d**) Vertical view of a supercell of the quasi-continuous phase-gradient metasurface.

**Figure A2:**
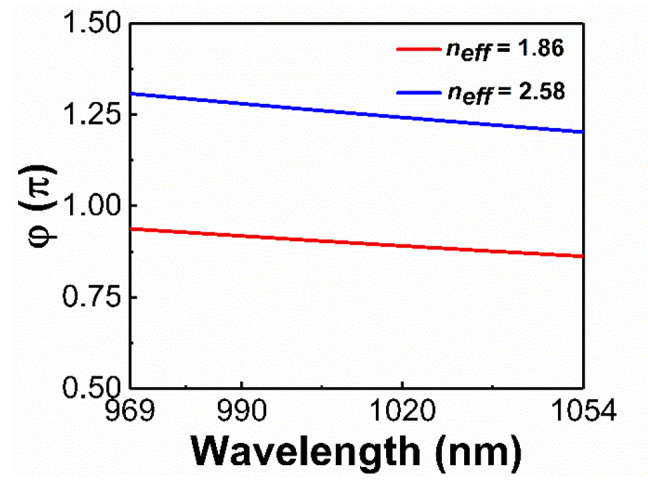
Within the wavelength range of 969 nm to 1054 nm, the calculated propagation phase is shown for the effective refractive index of 1.86 and 2.58, respectively.

## Data Availability

Data sharing is not applicable to this article.

## Appendix A. Design of the Trapezoidal Antenna I

To design the quasi-continuous trapezoid-shaped antennas and explain the phase response of the quasi-continuous metasurface, the trapezoidal nanoantenna can be approximately viewed as composed of multiple discrete geometry-varied blocks [28]. We simulate a simple homogeneous metasurface composed of rectangular silicon blocks rested on silica film for the design, as shown in Figure A1a. The period of the unit cell is P = 190 nm, and the height of the silicon block h is 240 nm. The lengths of the block in the x- and the y-directions are the same. The phase of the transmitted light as the function of the width W of the silicon block at the wavelength of 694.3 nm is depicted in Figure A1b. We find that a full 2π phase shift can be achieved when the width of the block changes from 50 nm to 180 nm. Based on this, we chose eight different blocks with π/4 phase increments to cover a 0~2π phase shift as shown in Figure A1c. The widths of these blocks were 66 nm, 94 nm, 109 nm, 120 nm, 131 nm, 145 nm, 163 nm and 176 nm, respectively. When combining these nanoblocks into a trapezoidal antenna, considering the introduction of another array with opposite spatial arrangement in the next step, the length of the trapezoidal antenna cannot be too large. Therefore, we arranged these blocks compactly from small to large, instead of filling the gaps between them as depicted in Figure A1d. In this process, to obtain the standard trapezoidal structure and gain a good optical performance, we optimized the geometrical parameters of the trapezoidal antenna as Px = 1360 nm, Py = 190 nm, L = 806 nm, W_1_ = 66 nm, W_2_ = 154 nm.

## Appendix B. Unequal-Power Beam-Splitting Performance of Antenna I Array Under x-Polarized Incidence

As mentioned above, when x-polarized light with a wavelength from 969 nm to 1054 nm is incident on the antenna I array, a phase profile of 0-π-0 is approximately formed over a period of this array, which causes the outgoing light to propagate along the direction of −1 and +1 diffraction orders, but the intensity of these two beams is different [22,23]. This phenomenon can be explained by effective medium theory and transmission phase theory. According to the effective medium theory, for x-polarized incidence, the effective refractive index of rectangular structure can be expressed as:

(4) $$n_{eff}=\sqrt{f{\left(n_{1}+k_{1}\right)}^{2}+(1-f){\left(n_{2}+k_{2}\right)}^{2}}$$

where *f* is the duty cycle of the rectangular grating, and *n* and *k* are the real and imaginary parts of the refractive index, respectively. Subscripts 1 and 2 represent the material of the rectangular structure and the surrounding medium of the rectangular structure, respectively. Therefore, *n*_2_ = 1, *k*_2_ = 0. Therefore, Equation (4) can be simplified as:

(5) $$n_{eff}=\sqrt{f{\left(n_{1}+k_{1}\right)}^{2}+(1-f)}$$

In order to simplify the calculation, we regard the refractive index of silicon material as a fixed value of 3.57 [27] in the wavelength range of 969 nm to 1054 nm, and set the imaginary part of the refractive index to 0. For a trapezoidal structure, the calculation of the duty cycle is complicated. We divide the trapezoidal nanoantenna into several nanoblocks to approximately calculate the change of trapezoidal effective refractive index along the x-direction. Here, to ensure that the effective medium theory is applicable, the trapezoidal antenna is divided into as many nanoblocks as possible. The length *L_i_* of each nanoblock along the x-axis is (L/i) nm. The duty cycle of the block can be calculated by $f=(W_{i}\times L_{i})/(P_{y}\times P_{x}{}_{i})=[W_{i}\times (L/i)]/[P_{y}\times (P_{x}/i)]=(W_{i}\times L)/(P_{y}\times P_{x})$. Among these blocks, the smallest one has a width of 66 nm and a duty cycle of 0.21. The largest one has a width of 154 nm and a duty cycle of 0.48. According to Equation (5), the effective refractive index of the smallest and the largest block is 1.86 and 2.58, respectively. According to the propagation phase theory [30], the relationship between the propagation phase and the effective refractive index is φ = h × 2π × *n_eff_*/λ_0_. Based on this formula, in the wavelength range from 969 nm to 1054 nm, we calculate the propagation phase with an effective refractive index of 1.86 and 2.58, respectively, as shown in Figure A2. When the effective refractive index is 1.86, the value of the abrupt phase induced by the antenna I changes from 0.85π to 0.92π. For the effective refractive index of 2.58, the value of the abrupt phase induced by the antenna I changes from 1.17π to 1.28π. According to the above analysis, we approximate that the effective refractive index of antenna I changes from 1.86 to 2.58 along the x-direction. The phase delay caused by antenna I along the x-direction is variable, but it can always achieve the abrupt phase close to π. Thus, the antenna I array can exhibit a broadband unequal-power beam-splitting performance when x-polarized light is incident. Here, we qualitatively explain the physical mechanism of broad-band unequal-power beam-splitting, and the specific optical response depends on further calculation using the simulation.
